# Life-history strategies in zooplankton promote coexistence of competitors in extreme environments with high metal content

**DOI:** 10.1038/s41598-018-29487-3

**Published:** 2018-07-23

**Authors:** Adriana Aránguiz-Acuña, Pablo Pérez-Portilla, Ana De la Fuente, Diego Fontaneto

**Affiliations:** 1 Faculty of Sciences, Chemistry Department, Universidad Católica del Norte, Angamos 0610, Antofagasta, Chile; 2 National Research Council of Italy, Institute of Ecosystem Study (CNR-ISE). Largo Tonolli 50, I-28922 Verbania, Pallanza (VB) Italy; 3 Centro de Investigación Tecnológica del Agua en el Desierto (CEITSAZA), Universidad Católica del Norte, Casilla 1280 Antofagasta, Chile

## Abstract

The toxicity of pollutants on aquatic communities is determined by the specific sensitivities and by the ecological relationships between species, although the role of ecological interactions on the specific sensitivity to pollutants is complex. We tested the effect of exposure to copper on the life-history strategies of two coexisting rotifer species of the genus *Brachionus* from Inca-Coya lagoon, an isolated water body located in Atacama Desert. The experiments looked at differences in the response to the stress by chemical pollution mimicking field conditions of copper exposure, levels of food, and salinity, between single-species cultures and coexisting species. Under single species cultures, *B*. ‘Nevada’ had lower densities, growth rates, and resting eggs production than *B*. *quadridentatus*; when in competition, *B*. ‘Nevada’ performed better than *B*. *quadridentatus* in most life-history traits. *B*. ‘Nevada’ was a copper-tolerant species, which outcompeted *B*. *quadridentatus*, more copper-sensitive, with higher levels of copper. Species-specific responses to environmental conditions and pollution, plus differential relationships between population density and production of resting eggs, resulted in reduced niche overlap between species, allowing stabilized coexistence. The extreme environmental conditions and the isolation of the Inca-Coya lagoon, make it an excellent model to understand the adaption of aquatic organisms to stressed environments.

## Introduction

One pervasive signature of this era dominated by anthropogenic activities is the increasing release and accumulation of chemical pollution in the environment^1^, notwithstanding that its dangers are known since two centuries ago^2^. Biodiversity loss is occurring at unprecedented rates, and has been cited as a consequence of widespread anthropogenic environmental changes, including chemical pollution^3,4^. Many aquatic inland environments are hotspots of biodiversity, but are highly endangered because human demands on freshwater ecosystems have risen steeply over the past century leading to large and growing threats to their biodiversity around the world^5^.

The toxic effect of chemical pollutants in aquatic communities is determined by the inherent sensitivities of the single species present in the community and by the ecological relationships between them^6,7^. Moreover, the role of ecological interactions on the sensitivity of each species when exposed to the toxic effects of pollutants is complex. Greater toxic effects of pollutants are often found in laboratory experiments on interacting populations than on single species^8,9^, even if interspecific competition is known to reduce the toxic effect of pollutants at the community level^10–12^. Therefore, complex response of interacting species are expected in natural polystressed environments.

Our aim is to test if and how exposure to chemical pollution modifies the competitive output of coexisting closely related species, trying to understand the potential life-history strategies allowing coexistence during the stress by pollution. To achieve such goal, we used carefully controlled experimental settings on laboratory cultures originating from the environment, mimicking the toxicant concentrations and other environmental variables actually experienced by those organisms in the field. As a model system we selected two coexisting species of planktonic rotifers of the same genus, living in a remote and isolated small lake, the Inca-Coya lagoon in the Atacama desert in Chile, where levels of heavy metals are naturally high, and raising because of nearby mining activities^13^.

Zooplanktonic communities have been used as model systems to study the process that maintain biological diversity^14,15^: life history traits in zooplankton allow trophic differentiation^16,17^ and spatial diversification of niches^18,19^. Moreover, temporal shifts and local coexistence of competing species can be observed in zooplankton species because of substitution processes in the water column, locally surviving adverse conditions through the production of latent forms. Thus, the response to metal pollution for planktonic rotifers under competition in Inca-Coya lagoon can be mediated by a change in reproductive mode and by differential investment in diapause production.

In monogonot rotifers, as those of the genus *Brachionus*, there is a hetereogonic reproductive cycle. They have a dominant parthenogenetic phase, when individuals reproduce clonally, and a sexual phase (mixis), in which, after the appearance of males, sexually inseminated females produce diapausing embryos called resting eggs^20–22^. The temporal compromise between rapid population growth by parthenogensis and population collapse during the sexual cycle suggests a loss of competitive ability through time^23,24^, but, in turn, allows the population persistence through resting eggs during periods of adverse conditions^25^. Therefore, a population that is producing diapausing forms may be temporally excluded from the water column opening the opportunity for other competing species that do not produce resting eggs at the same time.

In addition, *Brachionus* rotifers are frequently used in ecotoxicologal bioassays^26,27^. The effects of toxicants as heavy metals on the life cycle and on sexual reproductive parameters are known for *Brachionus* rotifers^28,29^. We previously showed that the shift from parthenogenesis to sexual reproduction or mixis with production of resting eggs is promoted in one species of *Brachionus* from Inca-Coya lagoon by experimental exposure to copper, suggesting that mixis and production of resting eggs could be an escape strategy^29^. Such biological attributes allow *Brachionus* to be reliable model systems to test the effects of pollution on life history strategies of competing populations of coexisting species^30^.

Here, we looked for mechanisms allowing species coexistence under environmental stress, mediated either by differential tolerance to copper under different environmental conditions, or by differential investment in resting eggs. We specifically tested if more sensitive species to copper are more prone to invest in resting eggs production than more tolerant species, and if these make a lower investment in diapause. We tested this hypothesis by exposing lab cultures of the two species to different levels of copper addition, under a combination of different levels of food and salinity with and without the effect of competition, mimicking the potential levels of food, salinity, and copper found in the field. We analyzed the response of the different lab conditions to demographic parameters of reproductive success, together with the production of resting eggs.

In order to be able to perform reliable inference from our lab experiments, we also tested several assumptions for the conditions for coexistence of the species, namely whether (1) tolerance to copper and (2) ability to resource exploitation calculated as the clearance rates on microalgae were similar in the two species, (3) if differentiation of their niches in relation to salinity was a major abiotic factor, and (4) if food level was a major biotic factor with similar responses.

## Methods

### Isolation of rotifers

Lab cultures were started from single resting eggs collected in the superficial sediment of Inca-Coya lagoon, Chile (22°20′S-68°35′W), a small lake located at Chiu-Chiu village at 2534 m.a.s.l. in the Atacama Desert. Mean annual precipitation is 6.1 mm and nearly 90% of the annual rainfall is recorded in the rainy season called “Altiplanic Winter”^31^, which occurs in the southern hemisphere summer. The extremely arid conditions and high evaporation (between 2000 and 3000 mm·year^−1^) maintain high concentrations of arsenic, copper, boron, chloride, sulfate, and other chemicals throughout the water bodies of the region^32^ and broad salinity values ranging from less than 0.5 g·L^−1^ to more than 10 g·L^−1^^13,33^. Data obtained from Inca-Coya water column at different year seasons, show pH levels ranged from 9.4 at surface to 10 at bottom, and concentrations of total copper in water around 50 mg·L^−1^ (personal observations).

Sediment samples were collected from the deepest part of Inca-Coya lagoon (18 m depth). Samples were stored at 4 °C and dark conditions until they were processed. Resting eggs were isolated from the surface sediments using a sugar flotation technique^34,35^. Resting eggs of rotifers of the genus *Brachionus* were individually isolated in 96-multiwell dishes and induced to hatch at 20 °C under white fluorescent constant illumination (150–170 μmol quanta m^**−**2^ s^**−**1^). F/2 medium^36^ was prepared with diluted artificial seawater (Instant Ocean^TM^, Aquarium Systems) with 2.5 g L^−1^ salinity. Dishes were checked every 24 h for hatchlings for up to 15 days. Clonal cultures were obtained by asexual proliferation of individual females hatched from isolated resting eggs.

Stock rotifer cultures from isolated clones were maintained at the same conditions that were used during hatching. Cultures were fed daily with the green microalga *Nannochloropsis gaditana* at a density of 1 × 10^6^ cell mL^−1^, which was cultured with f/2 medium. The algae used as food were harvested during the exponential growth phase, centrifuged at 3000 rpm for 5 minutes and re-suspended in distilled water for use. Their concentration was measured by direct counting. The medium with concentrated microalgae was renewed every two days.

### Species identification

The two most common and abundant planktonic rotifers in the lagoon are known to be of the genus *Brachionus*, namely *B*. *plicatilis* s.l. and *B*. *quadridentatus* s.l. (personal observation); individuals of these two species were selected for the experiments. The *B*. *plicatilis* complex is a collective name for at least 15 species with different ecological features and that are difficult to identify by morphology only^37,38^. Thus, we used a mitochondrial and nuclear DNA taxonomy approach to identify which of the known species in the *B*. *plicatilis* complex was present in the lagoon and was used for the experiments. Recent studies on *B*. *quadridentatus* suggest that also this is a complex of different species, even if the picture is not yet so clear as in the previous species complex^39^; we thus here report DNA sequence information also for the clones of this species used in the experiment, to allow future researchers to understand which of the cryptic species in the complex was used.

DNA extraction was performed from single eggs and individual rotifers using Instagene Chelex Matrix (BioRad). A fragment of the mitochondrial gene cytochrome *c* oxidase subunit I (COI) (ca. 661 bp) was amplified using the LCO1490 and HCO2198 primers^40^. PCR was performed with 5 µL of DNA of each analyzed clone in a total volume of 50 µL. The thermal profile consisted of a 3 min initial cycle at 93 °C, followed by 40 cycles of 92 °C for 15 s, 50 °C for 20 s, 70 °C for 1 min, and with a final extension of 72 °C for 3 min^37^. Each PCR product was checked in agarose gel. Purification and sequencing were performed in ABI Prism 3500 xl Applied biosystems, equipment in both direction. The chromatograms were checked in Chromas 2.6 program (http://technelysium.com.au/wp/chromas/). For the sequences of the *B*. *plicatilis* complex, BLAST searches in the NCBI database were used to check the closest similarity to the different species of the *B*. *plicatilis* and assign the lab cultures to one of the 15 species identified by Mills *et al*.^38^. For the *B*. *quadridentatus* complex, we simply checked in BLAST whether the organisms were of this species complex, and which known sequences were the most similar, because no clear taxonomic revision has been performed on the complex yet.

For the *B*. *plicatilis* species complex, we also amplified a fragment of the nuclear Internal Transcribed Spacer 1 (ITS1) from the same animals used to amplify COI, in order to confirm the species identity. ITS1 was amplified using the III R and VIII F primers^41^. PCR was performed with 5 µL of DNA of each analyzed clone in a total volume of 50 µL. The thermal profile consisted of a 3 min initial cycle at 93 °C, followed by 40 cycles of 92 °C for 15 s, 50 °C for 20 s, 70 °C for 1 min, and with a final extension of 72 °C for 3 min^37^.

### Copper toxicity tests

Toxicity bioassay tests were conducted to assess the similarity of the different clones of the two species in their tolerance to copper, one of the metals found in high concentrations in Inca-Coya pond.

Standard acute bioassays were conducted for 48 h following the guidelines described by ASTM^42^. Neonates (<6 h-old) were selected from both species for the bioassays. After conducting preliminary bioassays, ultimate bioassay was conducted at 0, 0.08, 0.16, 0.4, 1, 2 and 4 mg L^−1^ of CuSO_4_·5·H_2_O for *B*. *plica*tilis and 0, 0.01, 0.02, 0.04, 0.08, 0.16, 0.4 and 1 mg L^−1^ for *B*. *quadridentatus*. These concentrations were tested because full mortality was obtained at different range of concentrations for both species. In order to control for addition of sulphates on the mortality estimation caused by exposure to metals, additional bioassays were conducted with MgSO_4_. These were performed under identical conditions and with the same concentrations of sulphates employed in the copper test described above. The difference in the mortality of rotifers between treatments with copper and magnesium sulphate was estimated to identify toxicity of copper on rotifer.

Concentration-response curves and effective concentrations (ECx) of copper were obtained by a non-linear regression on survival data, and calculated in the package drc^43^ in R 3.3.3^44^. Results are showed as response ratio (dead individuals at the end of the experiment/total individuals at the start of experiment) over CuSO_4_ concentration (mg·L^−1^) in logarithmic scale. Student’s t-test was used to compare EC_50_ values between species. From these results, a copper sub-lethal concentration for both species was selected, to be used in the following experiments.

### Clearance rates

In order to identify the ability of competitive exploitaton of both species, their feeding behavior was studied by measuring clearance rates in short-term feeding experiments in monoalgal cultures of *Nannochloropsis gaditana* following Ciros-Pérez *et al*.^45^. Fifteen clones of each *Brachionus* species proliferated from single hatchlings were founded and kept individually in 20 mL vessels at 22 °C and 5 g L^−1^ salinity. These clonal linages were maintained isolated under same conditions described in ‘Isolation of rotifers’ section. Previous to experiments, multi-clonal pre-experimental populations were established by mixing 25 females of each clone^46^ to obtain a pre-experimental population of 25 × 15 = 375 individuals for each species. The rotifers were transferred from the pre-experimental cultures to the experimental food concentration 1 h before the experiments. For that purpose, these cultures were filtered through a 50 µm mesh, and the retained rotifers washed with saline water at 5 g L^−1^ to eliminate any remnants of algae. Afterwards, 20 rotifers from the multi-clonal culture were transferred to a well from multiwell plates containing 2.5 mL of culture medium with the experimental concentration of algae of 1 × 10^6^ cells·mL^−1^. The wells were kept for 10 hour in a shaker at a constant speed (6 rpm), at 20 °C, and in darkness to avoid algal growth during the experiment. After that time, the wells were fixed with 20 µL of Lugol’s solution. Six replicates were performed for each rotifer species. Additionally, six wells with microalgae without rotifers were used as controls, three of them were fixed immediately after inoculation with the algae and other three were fixed at the end of the experiment. The final algae concentration was assessed spectrophotometrically at 540 nm. The clearance rates, CR, were calculated following Peters^47^:

$$CR=\frac{ln{C}_{{\rm{o}}}-ln{C}_{t}}{N\times t},$$

where *C*_0_ and *C*_t_ are the initial and final algae concentrations, respectively; *N* is the rotifer density and *t* is the times in hours. *C*_0_ value was estimated as the average concentration of the three control tubes. Student’s t-test was used to compare CR values between species on six replicates for each species.

### Interspecific competition experiments

Experimental populations of both species were grown separately and in competition under different experimental conditions. Experiments were performed under three different salinities (2.5, 5 and 10 g·L^−1^), two exposure conditions to CuSO_4_·5·H_2_O (without and with copper in sublethal concentration) and two food levels (low: 2.5 × 10^5^ cells·mL^−1^, and high: 1 × 10^6^ cells·mL^−1^). Thus, the experiment consisted of 108 cultures (two species in monospecific cultures or in competition × three salinities × two copper exposure × two food levels × three replicates). Single-species treatments were initiated with 0.5 ind·mL^−1^ rotifer densities, while competing-species treatments were initiated with 0.25 ind·mL^−1^ of each species, in order to maintain the same total density of rotifers in all the treatments. Experimental treatments were initiated with individuals coming from acclimation cultures to corresponding salinity. The experiment was conducted in 200 mL of medium in 500 mL glass containers, under dark conditions to avoid the algae proliferation, temperature of 22 ± 1 °C, and continuously shaken at low speed (40 rpm). Every two days, three aliquots of 20 mL from each replicate were isolated to count numbers of: asexual females, sexual females, unidentified females (without eggs), males, and resting eggs produced. Experiments were finished after 30 days. From these data we evaluated the total population density as the sum of all individuals counted along experiment on each replicate. We then calculated the observed growth rates as ${r}_{obs}=\frac{\mathrm{ln}({N}_{t}/{N}_{0})}{t}$, where *N*_*t*_ and *N*_0_ are number of females at the beginning and after the period of initial exponential growth. The exponential growth phase was identified for each time series by maximizing the explained variance of a linear regression of ln(*N*) versus time. Additionally, the potential growth rate (*r*_*pot*_) was calculated, defined as the growth rate that population would have if all of its females were reproducing asexually^48^, and total resting eggs produced by treatment.

The effect of copper, under different salinities and with different levels of food resources interacting with competition, was tested on four demographic life history parameters: population density (density), observed growth rates (r_obs_), potential growth rate (r_pot_), and total number of resting eggs (eggs) for both species.

For each of the four life history parameters used as response variables (density, r_obs_, r_pot_, eggs) in each species, we first tested the main effect of all explanatory variables (copper, salinity and food) and their interactions on the single-species cultures, and then explored the differences in the experiments in presence of competitor (“Competition” model) and eliminating the effect of the presence of the competitor (“Only under competition” model) for each of the species. This model was adjusted to explore if the presence of the competitor modified the effects of the other factors and the observed interactions in the competition treatment. In all cases, linear models were fitted, on the raw values for density and log transformed values for the other three response variables. Model fit was checked through normality of residuals, QQ plots, and Cook’s distances^49^. Moreover, given the inherent complexity of the models with fixed terms and their interactions, we used a model averaging approach to identify significant predictors and estimated also their relative importance value, as the sum of the Akaike weights (cumulative AIC) of the sub-models in which the variable appears, ranging from 0 (=no importance) to 1 (=high importance)^50^. In the graphs, we used the results of Holm-Sidak tests to mark significant differences between population densities of both species reached in control and copper addition treatments. All models were fitted in R, and model averaging was performed in the R package MuMIn 1.15–6^51^.

Comparisons in life history traits between species were performed with the same statistical approach: linear models including all relevant predictors and their interactions, followed by multimodel averaging. In addition, Pearson’s correlation tests were used to check for correlation between life history demographic parameters, regardless of the environmental drivers of the differences between them.

## Results

### Species identification

A total of 50 resting eggs collected in the sediments of Inca-Coya lagoon were morphologically identified as belonging to the genus *Brachionus*. Depending on their size and shape 25 of them were provisionally identified as *B*. *plicatilis* s.l. and 25 as *B*. *quadridentatus* s.l. The 50 resting eggs were all used to start clonal cultures and all clones of both species were maintained successfully as stock cultures in laboratory conditions. The clones of *B*. *plicatilis* s.l. belonged to three cytochrome c oxidase I (COI) haplotypes (GenBank Accession number: KU299431.1, DQ664507.1, KU189744.1) with uncorrected genetic distances between them ranging from 0.1% to 1.4%. The most closely related species within the *B*. *plicatilis* complex, with a distance between 10.3% and 20.1%, is in the group of so-called large *B*. *plicatilis* group, informally known as *B*. ‘Nevada’ according to Gómez *et al*.^37^ or L4 according to Mills *et al*.^38^. The cultures revealed no variability in the Internal Transcribed Spacer 1 (ITS1) fragment, with all the clonal cultures (GenBank Accession numbers: LC339820.1) being identical between themselves, and to the ones already known for the still undescribed *B*. ‘Nevada’ or L4^38^.

The clones of *B*. *quadridentatus* s.l. belonged all to the same COI haplotype (GenBank Accession number: AF387294.1) with uncorrected genetic distances of 14.1% to the closest GenBank hit of sequences of the same morphological species. The two species will then called *B*. ‘Nevada’ and *B*. *quadridentatus* throughout the paper.

### Toxicity bioassays and clearance rates

**Figure 1:**
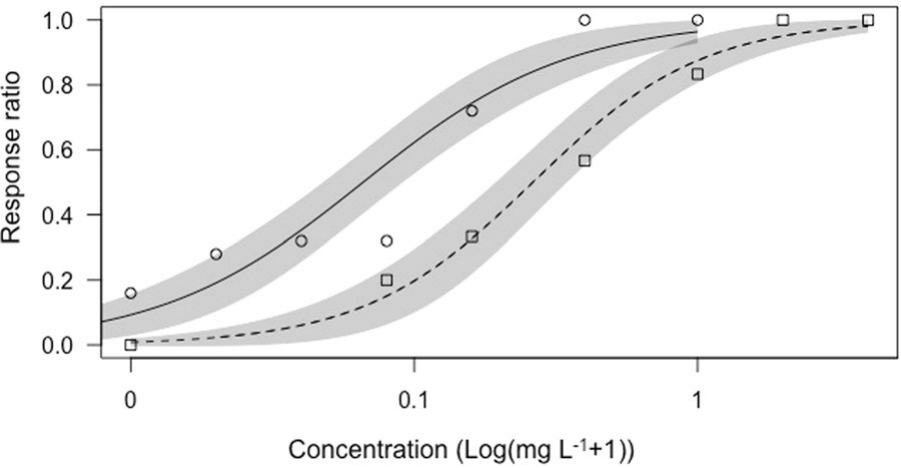
Dose-response curves of *B*. ‘Nevada’ (dashed line) and *B*. *quadridentatus* (continuous line) experimental populations from Inca-Coya lagoon to CuSO4 concentrations (mg·L^−1^) in logarithmic scale. 95% Confidence Intervals are shown in grey shaded areas.

Significantly higher tolerance to copper was observed in *B*. ‘Nevada’ than in *B*. *quadridentatus* from sediments from Inca-Coya pond (t-test, t_8_ = −4.908, P < 0.001; Fig. 1). A concentration of 0.05 mg·L^−1^ of this metal, corresponding to effective concentration 10% (EC_10_) for *B*. ‘Nevada’ and 40% (EC_40_) for *B*. *quadridentatus*, was selected as sublethal for both species, and was used in the experiments with exposure to copper.

No significant differences were observed between feeding rates of *B*. ‘Nevada’ (4.55 ± 0.04) and *B*. *quadridentatus* (4.74 ± 0.17) (t-test, t_5_ = −0.848, P = 0.435) suggesting equivalence in filtration abilities of both species on the alga *N*. *gaditana*.

### Single-species responses

**Figure 2:**
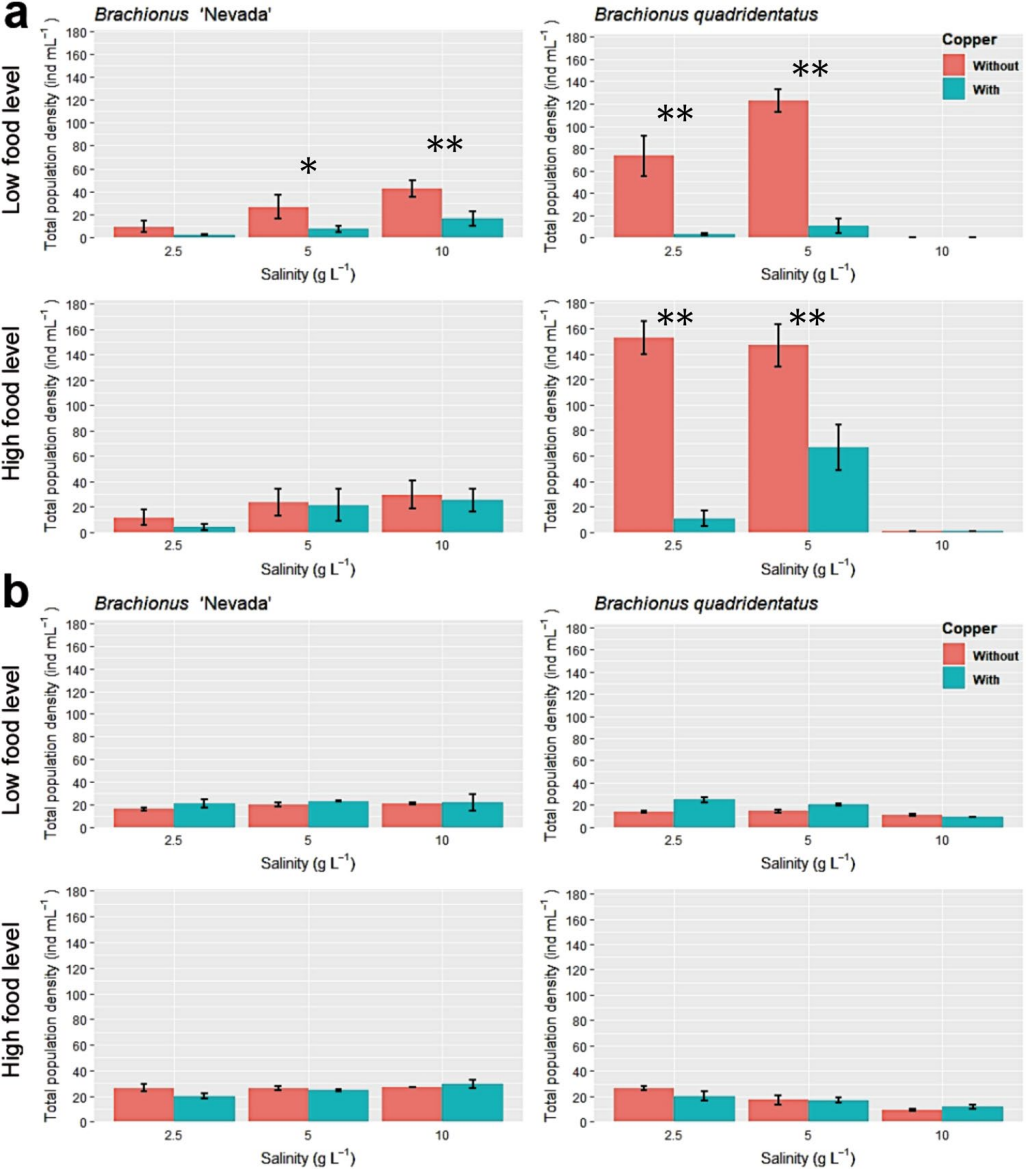
Total population density reached in (**a**) single-species treatments and (**b**) competition treatments, by *B*. ‘Nevada’ (left) and *B*. *quadridentatus* (right) under conditions of low (above) and high (below) food levels, range of salinities, in the control conditions and when exposed to copper. Mean values and standard deviations are shown. Asterisks represent significant differences between control and copper treatment (Holm-Sidak test, P < 0.002*, P = 0.000**).

*Brachionus* ‘Nevada’ grew well under all conditions and its population densities were positively explained by salinity values (Fig. 2, Table 1, Table S1A). Growth rates, expressed as observed growth rates (r_obs_) and potential growth rates (r_pot_), were positively explained by salinity too, and negatively by copper (Table 1, Table S1A). The correlation of the production of resting eggs were not so clear, and almost any environmental change that was tested affected them: copper and food positively, salinity negatively (Table 1, Table S1A). The production of resting eggs was significantly negatively correlated to density (Pearson’s r = −0.54, t_70_ = −5.4, P < 0.0001).

**Table 1:** Summary of significant effects detailed in Supplementary Tables S1–S3, obtained from lineal models adjusted for life-history traits: density, *r*_*obs*_, *r*_*pot*_ and resting eggs produced by *B*. ‘Nevada’ and *B*. *quadridentatus*.

Model	Predictor	*B*. ‘Nevada’				*B*. *quadridentatus*			
		Density	r_obs_	r_pot_	Eggs	Density	r_obs_	r_pot_	Eggs
Single-species	Copper		−	−	+	−			−
	Food				+				
	Salinity	+	+	+	−	−	−	−	−
	Copper × Salinity				−	+			+
	Food × Salinity				+				
Competition	Competition	+	+	+	−	−	−	−	−
	Copper		−	−	+	—			−
	Food					+			
	Salinity	+	+	+	−	−	−	—	−
	Competition × Copper				−	+			—
	Competition × Salinity	−			−	+	+	+	+
	Copper × Salinity					+			+
	Competiton × Copper × Salinity				+	−			−
Only under competition	Copper		−	−		−	−		
	Food	+				+	+	+	
	Salinity			+		−	-	−	
	Copper × Food					+	+	+	
	Copper × Salinity					+	+		
	Food × Salinity					+	+		
	Copper × Food × Salinity					−	−		

Factors of copper addition, salinity and food levels, were tested when species grew in monocultures, under competition and only under competition with the other species. Plus sign: positive effect, minus sign: negative effect.

*Brachionus quadridentatus* had a different response to the tested factors: it did not grow at all at higher salinities (Fig. 2), its population densities and its production of resting eggs were positively correlated (r = 0.92, t_70_ = 19.4, P < 0.0001) and negatively influenced by copper and by salinity. Its growth rates were negatively influenced by salinity (Table 1, Table S1B).

When comparing the two species under single-species lab cultures, all of the four life history traits resulted different between the two species, with *B*. ‘Nevada’ having invariably lower densities, r_obs_, r_pot_, and number of resting eggs than *B*. *quadridentatus* under the different treatments, even in cases when a significant interaction with copper or salinity was found (Table S4).

### Interspecific competition experiments

Competition had a positive effect on *B*. ‘Nevada’ for life history traits except production of resting eggs and a negative effect on *B*. *quadridentatus* for all four life history traits (Table 1, Table S2). The effect of copper in *B*. ‘Nevada’ was negative for r_obs_ and r_pot_, but positive for the number of resting eggs, similarly to the single-species experiments without competition (Table 1, Table S1A); in *B*. *quadridentatuts* the effect of copper was negative for density and resting eggs, similarly to the experiments without competition (Table 1, Table S1B), even if a significant interaction was found for this species between the effects of copper and competition.

Analyzing life history traits of each of the two species only when under competition, copper was confirmed as a negative variable for growth rates in *B*. ‘Nevada’ (Table 1, Table S3A), whereas for *B*. *quadridentatus* the effect of copper was negative and strongly interacted with food and salinity for densities and growth rates (Table 1, Table S3B). Interestingly, the production of resting eggs was not affected by any variable when this species was under competition with *B*. ‘Nevada’. When comparing the two species under competition, no differences between them were present in density or growth rates, and the only difference was in the number of resting eggs, with *B*. *quadridentatus* producing more resting eggs than *B*. ‘Nevada’ (Table S5).

## Discussion

Coexisting rotifer species from Inca-Coya lagoon responded dissimilarly to copper, a metal present in high concentrations in the sediments and in the water column of the lagoon. Copper affected both species, diminishing population densities. Nevertheless, the magnitude of this response was higher for *B*. *quadridentatus*, the most sensitive species, than for *B*. ‘Nevada’, which resulted more tolerant to copper. The main result of our study suggests that coexistence is possible because the two species are differentially adapted to environmental conditions. When in single-species cultures, *B*. ‘Nevada’ had lower densities, r_obs_, r_pot_, and number of resting eggs than *B*. *quadridentatus* under the different treatments; yet, when in competition, the higher tolerance to copper of *B*. ‘Nevada’ makes this species a better competitor. Competition had a negative effect on *B*. *quadridentatus* for all four life history traits and a positive effect on *B*. ‘Nevada’ for all life history traits (except for the production of resting eggs). Our results could support the idea that competition alters the response to copper and allows a copper-tolerant species to coexist with a copper-sensitive species in a complex scenario of differential responses to environmental variables such as salinity and food availability.

Our main hypothesis was that most copper-sensitive species (in our case *B*. *quadridentatus*) would be more prone to invest in resting eggs production than the most copper-tolerant species (in our case *B*. ‘Nevada’). We did not find such a clear pattern, because the two species had a differential density-dependent effect on the production of resting eggs: whereas in *B*. ‘Nevada’ the production of resting eggs was linked to population decline, in *B*. *quadridentatus* the production of resting eggs was positively correlated to population density. The production of resting eggs in both species was a sensitive life-history trait to exposure to copper. Nevertheless, the two species were impacted in opposite directions by copper: the production of resting eggs was favored in *B*. ‘Nevada’, whereas it was negatively affected in *B*. *quadridentatus*. In many monogonont rotifer species the beginning of sexual reproduction, and the subsequent production of resting eggs is density-dependent, because it is triggered by a quorum-sensing molecule produced by the females themselves^25,52^. In addition, other factors, such as salinity, temperature, and food availability, could also have an impact on production of resting eggs^53,54^.

Salinity was a main abiotic factor differentially affecting the two rotifer species. Salinity is known as a relevant driver of density and composition of zooplankton communities and populations of inland aquatic ecosystems^55–57^. In general, higher salinities are stressful^58^, and there is an inverse relationship between salinity and zooplankton richness and density^55^. The two species had a different response to the salinity values experienced in the field in Inca-Coya lagoon: *B*. ‘Nevada’ was positively affected by higher salinity, whereas *B*. *quadridentatus* did not survive at all at the highest salinity; the production of resting eggs was consistently negatively affected by salinity. Galbaldón *et al*.^59^ showed that salinity was pivotal to allow coexistence of closely related species of the genus *Brachionus*: fluctuating salinity allowed changes in dominance of one species over the competitor.

The tested food levels, similar to those found in the field in Inca-Coya lagoon, did not have any effect on the response of the two species. Feeding abilities, expressed as clearance rates, suggested exploitative equivalence between species, even if competition had a greater negative impact on *B*. *quadridentatus* than on *B*. ‘Nevada’.

Overall, the results of our experiments allowed us to suggest that the copper-tolerant rotifer species may be a short-term dominant competitor at higher levels of copper but could be more affected in its long-term survival parameters in interspecific interaction scenario, especially because the production of resting eggs was connected to population decline, with living animals disappearing from the water column.

Trade-offs are important in life-history evolution and coexistence of competitors. In rotifers of the genus *Brachionus*, a trade-off is known between the rapid population growth during the parthenogenetic phase, when individuals reproduce clonally, and the interruption of population growth during the sexual phase. This phase is metabolically and demographically costly, due to the appearance of males, to the production of resting eggs and to the (complete or partial) disappearance of parthenogenetic females^20–22^. The temporal compromise between colonization of the water column by population growth via female parthenogenesis and the production of resting eggs during the sexual cycle suggests a loss of competitive ability of the active population^23,24,60^. In our results, the exposure to copper increased the costs for *B*. *quadridentatus*, even if this species did not have any trade-off between population density and production of resting eggs.

Closely related rotifer species often coexist in temporal ponds characterized by environmental fluctuations. Nevertheless, many zooplankton species are temporary even in permanent ponds and lakes, as in case of Inca-Coya lagoon. Coexistence of closely related species (as those from the *B*. *plicatilis* complex) may be obtained by differential responses to environmental conditions such as salinity^61–63^, differential susceptibility to predation and/or resource partitioning^45,64,65^. Differences in species-specific responses to environmental conditions and pollution should result in reduced niche overlap between coexisting species to minimize the impact of fitness inequalities on competitive interactions, allowing stabilized coexistence^66–68^. Stabilizing coexistence describes differences in species-specific responses to varying environmental conditions that result in reduced niche overlap, thus minimizing the impact of fitness inequalities on competitive interactions^66–68^. The differential responses in rate of population growth and diapause production of the two coexisting species in Inca-Coya lagoon, interacting with increasing salinity and copper concentrations, might reduce the intensity of competition between them and promote their coexistence over time. Different strategies to face metal pollution may thus allow coexistence of rotifers. The long-term competitive output will rely on the pattern of production, viability and hatchability of resting eggs under a range of suitable conditions^59^, because competitive exclusion in temporarily active populations does not necessarily mean long-term exclusion^63^. Further investigations may be focused in assess the full contribution of resting eggs to populations fitness in long-term scenario in Inca-Coya lagoon.

Previously, we showed that copper negatively affected the hatching success of resting eggs and performance of hatchlings on the same population of *B*. ‘Nevada’ from Inca-Coya lagoon, with greater negative effects for resting eggs produced under non-metal conditions, suggesting an adaptive advantage of populations from natural sediments exposed to metals^69^.

There is particular concern that contamination of aquatic ecosystems may affect ecological functions in freshwater ecosystems^70^, which are also sensitive to the effects of multiple stressors^71^. Here, we addressed the interacting effects of copper, salinity, food, and competition on two naturally coexisting rotifer species of the genus *Brachionu*s and disentangled the single and multiple roles of each stressor; yet, other threats such as climate change could alter the observed response to historical exposure to metals depositions by changes in interspecific interactions. Novel combinations of stressors, such as the addition of temperature variation, may have serious consequences for biodiversity and ecosystem functioning^72^. For zooplankton, seasonal succession of competitors could be promoted by further variability in water temperature, through the development of ecological specialization^73^.

The extreme environmental conditions and the isolation of desert lagoons such as Inca-Coya lagoon make such sites highly vulnerable to increasing anthropogenic disturbance and place them at the focus of conservation studies; additionally, such peculiarities make them excellent models to study the adaption of aquatic populations to increasing harsh environments in a simplified ecological framework with strong stresses and few competing species.

## Electronic supplementary material

Supplementary tables
