# Supplementary material for: Life-history strategies in zooplankton promote coexistence of competitors in extreme environments with high metal content
Source: Sci Rep. 2018 Jul 23;8:11060. doi: 10.1038/s41598-018-29487-3 (PMC6056428; doi:10.1038/s41598-018-29487-3)
Supplement: Supplementary file 1 — Supplementary tables [file 41598_2018_29487_MOESM1_ESM.pdf]

# Life-history strategies in zooplankton promote coexistence of competitors in extreme environments with high metal content

Adriana Aránguiz-Acuña, Pablo Pérez-Portilla, Ana de La Fuente, Diego Fontaneto

Supplementary Table S1. Output of the linear regression models explaining the effect of copper, food, and salinity, in the single-species lab cultures, on the response of population density, $r_{obs}$, $r_{pot}$, and total resting eggs in the experiments for (A) *B.* 'Nevada' and (B) *B. quadridentatus*. Estimates, standard errors, z-values, and p-values are reported from multimodel averaging, together with relative importance values (RI). Significant predictors are marked in bold. Interaction terms with AICc<0.001 are not reported

A. *Brachionus* 'Nevada'

| Response | Predictor | Estimate | Standard Error | z | P | RI |
|---|---|---|---|---|---|---|
| Density | (Intercept) | 6.36 | 4.63 | 1.3 | 0.18 | |
| | Copper | -5.84 | 6.36 | 0.9 | 0.37 | 1.00 |
| | Food | -1.25 | 4.99 | 0.2 | 0.81 | 0.68 |
| | **Salinity** | **2.81** | **0.64** | **4.3** | **<0.00** | **1.00** |
| | Copper×Food | -6.57 | 7.91 | 0.8 | 0.41 | 0.55 |
| | Copper×Salinity | -0.32 | 0.74 | 0.4 | 0.68 | 0.34 |
| | Food×Salinity | 0.16 | 0.58 | 0.3 | 0.79 | 0.18 |
| | Copper×Food×Salinity | -0.08 | 0.56 | 0.1 | 0.88 | 0.03 |
| $r_{obs}$ | (Intercept) | 0.12 | 0.06 | 1.8 | 0.07 | |
| | **Copper** | **-0.24** | **0.10** | **2.5** | **0.01** | **1.00** |
| | Food | -0.00 | 0.03 | 0.1 | 0.95 | 0.30 |
| | **Salinity** | **0.02** | **0.01** | **2.2** | **0.02** | **1.00** |
| | Copper×Food | -0.01 | 0.03 | 0.2 | 0.86 | 0.08 |
| | Copper×Salinity | 0.02 | 0.02 | 1.2 | 0.23 | 0.73 |
| | Food×Salinity | 0.00 | 0.00 | 0.1 | 0.93 | 0.06 |
| | Copper×Food×Salinity | 0.00 | 0.00 | 0.0 | 0.98 | 0.00 |
| $r_{pot}$ | (Intercept) | 0.13 | 0.07 | 1.7 | 0.08 | |
| | **Copper** | **-0.23** | **0.11** | **2.1** | **0.04** | **1.00** |
| | Food | -0.01 | 0.04 | 0.1 | 0.90 | 0.33 |
| | **Salinity** | **0.03** | **0.01** | **2.5** | **0.01** | **1.00** |
| | Copper×Food | -0.02 | 0.05 | 0.3 | 0.77 | 0.13 |
| | Copper×Salinity | 0.01 | 0.02 | 0.9 | 0.38 | 0.58 |
| | Food×Salinity | 0.00 | 0.00 | 0.0 | 0.98 | 0.06 |
| | Copper×Food×Salinity | 0.00 | 0.00 | 0.0 | 0.99 | 0.01 |
| Eggs | (Intercept) | 41.16 | 4.06 | 9.7 | <0.00 | |

| | | | | | | |
|---|---|---|---|---|---|---|
| | **Copper** | **38.34** | **5.03** | **7.3** | **<0.00** | **1.00** |
| | **Food** | **27.83** | **5.03** | **5.3** | **<0.00** | **1.00** |
| | **Salinity** | **-3.91** | **0.59** | **6.4** | **<0.00** | **1.00** |
| | Copper×Food | -3.35 | 4.51 | 0.7 | 0.47 | 0.49 |
| | **Copper×Salinity** | **-3.92** | **0.68** | **5.5** | **<0.00** | **1.00** |
| | **Food×Salinity** | **2.90** | **0.68** | **4.1** | **<0.00** | **1.00** |

B. *Brachionus quadridentatus*

| Response | Predictor | Estimate | Standard Error | Z | P | RI |
|---|---|---|---|---|---|---|
| Density | (Intercept) | 207.46 | 19.87 | 10.1 | <0.00 | |
| | **Copper** | **-157.04** | **22.67** | **6.7** | **<0.00** | **1.00** |
| | Food | 54.57 | 26.59 | 2.0 | 0.04 | 1.00 |
| | **Salinity** | **-18.85** | **3.02** | **6.0** | **<0.00** | **1.00** |
| | Copper×Food | 5.17 | 17.59 | 0.3 | 0.77 | 0.24 |
| | **Copper×Salinity** | **15.07** | **3.32** | **4.4** | **<0.00** | **1.00** |
| | Food×Salinity | 4.30 | 4.07 | 1.0 | 0.30 | 0.67 |
| | Copper×Food×Salinity | -0.35 | 2.06 | 0.2 | 0.87 | 0.05 |
| $r_{obs}$ | (Intercept) | 1.09 | 0.12 | 9.1 | <0.00 | |
| | Copper | 0.01 | 0.07 | 0.2 | 0.85 | 0.29 |
| | Food | 0.01 | 0.07 | 0.2 | 0.86 | 0.28 |
| | **Salinity** | **-0.25** | **0.02** | **14.6** | **<0.00** | **1.00** |
| | Copper×Food | 0.00 | 0.03 | 0.0 | 0.97 | 0.02 |
| | Copper×Salinity | 0.00 | 0.01 | 0.1 | 0.96 | 0.06 |
| | Food×Salinity | 0.00 | 0.01 | 0.1 | 0.95 | 0.06 |
| $r_{pot}$ | (Intercept) | 1.11 | 0.12 | 9.2 | <0.00 | |
| | Copper | 0.02 | 0.08 | 0.3 | 0.80 | 0.30 |
| | Food | 0.01 | 0.07 | 0.2 | 0.88 | 0.28 |
| | **Salinity** | **-0.25** | **0.02** | **14.6** | **<0.00** | **1.00** |
| | Copper×Food | 0.00 | 0.02 | 0.0 | 0.98 | 0.01 |
| | Copper×Salinity | 0.00 | 0.01 | 0.1 | 0.94 | 0.06 |
| | Food×Salinity | 0.00 | 0.01 | 0.1 | 0.96 | 0.05 |
| Eggs | (Intercept) | 9.33 | 1.06 | 8.5 | <0.00 | |
| | **Copper** | **-7.20** | **1.40** | **5.0** | **<0.00** | **1.00** |
| | Food | 0.56 | 0.87 | 0.6 | 0.53 | 0.55 |
| | **Salinity** | **-0.86** | **0.15** | **5.5** | **<0.00** | **1.00** |
| | Copper×Food | -0.07 | 0.49 | 0.1 | 0.89 | 0.11 |
| | **Copper×Salinity** | **0.75** | **0.21** | **3.4** | **0.00** | **1.00** |

| | | | | | |
|---|---|---|---|---|---|
| Food×Salinity | 0.02 | 0.09 | 0.2 | 0.85 | 0.12 |
| Copper×Food×Salinity | 0.00 | 0.03 | 0.0 | 0.99 | 0.01 |

Supplementary Table S2. Output of the linear regression models explaining the effect of competition on the response of population density, $r_{obs}$, $r_{pot}$, and total resting eggs in the experiments, with different levels of copper, food, and salinity for (A) *B.* 'Nevada' and (B) *B. quadridentatus*. Estimates, standard errors, z-values, and p-values are reported from multimodel averaging, together with relative importance values (RI). Significant predictors are marked in bold. Interaction terms with AICc<0.001 are not reported.

A. *Brachionus* 'Nevada'

| Response | Predictor | Estimate | Standard Error | z | P | RI |
|---|---|---|---|---|---|---|
| Density | (Intercept) | 6.80 | 3.90 | 1.7 | 0.0857 | |
| | **Competition** | **17.58** | **5.12** | **3.4** | **0.0007** | **1.00** |
| | Copper | -5.68 | 5.24 | 1.1 | 0.2839 | 1.00 |
| | Food | -0.22 | 5.69 | 0.0 | 0.9695 | 0.99 |
| | **Salinity** | **2.63** | **0.55** | **4.7** | **0.0000** | **1.00** |
| | Competition×Copper | 2.45 | 6.82 | 0.4 | 0.7224 | 1.00 |
| | Competition×Food | -9.51 | 6.14 | 1.5 | 0.1253 | 0.89 |
| | **Competition×Salinity** | **-2.32** | **0.64** | **3.6** | **0.0004** | **1.00** |
| | Copper×Food | -6.59 | 8.32 | 0.8 | 0.4316 | 0.91 |
| | Copper×Salinity | 0.00 | 0.70 | 0.0 | 0.9952 | 0.58 |
| | Food×Salinity | 0.55 | 0.82 | 0.7 | 0.5099 | 0.51 |
| | Competition×Copper×Food | 14.75 | 8.47 | 1.7 | 0.0842 | 0.84 |
| | Competition×Copper×Salinity | 0.32 | 0.74 | 0.4 | 0.6643 | 0.25 |
| | Competition×Food×Salinity | -0.14 | 0.49 | 0.3 | 0.7861 | 0.14 |
| | Copper×Food×Salinity | -0.76 | 1.19 | 0.6 | 0.5269 | 0.34 |
| | Competition×Copper×Food×Salinity | 0.01 | 0.20 | 0.0 | 0.9619 | 0.01 |
| $r_{obs}$ | (Intercept) | 0.14 | 0.05 | 2.7 | 0.0062 | |
| | **Competition** | **0.13** | **0.05** | **2.5** | **0.0119** | **1.00** |
| | **Copper** | **-0.29** | **0.07** | **4.2** | **<0.0001** | **1.00** |
| | Food | -0.00 | 0.05 | 0.0 | 0.9550 | 0.65 |
| | **Salinity** | **0.02** | **0.01** | **2.6** | **0.0103** | **1.00** |
| | Competition×Copper | -0.03 | 0.05 | 0.5 | 0.5909 | 0.41 |
| | Competition×Food | 0.02 | 0.05 | 0.3 | 0.7604 | 0.30 |
| | Competition×Salinity | 0.00 | 0.01 | 0.1 | 0.8919 | 026 |
| | Copper×Food | 0.00 | 0.03 | 0.1 | 0.8829 | 0.16 |
| | Copper×Salinity | 0.02 | 0.01 | 2.2 | 0.0263 | 0.95 |
| | Food×Salinity | 0.00 | 0.01 | 0.3 | 0.7618 | 0.25 |
| | Competition×Copper×Food | 0.00 | 0.01 | 0.1 | 0.9439 | 0.01 |

| | | | | | | |
|---|---|---|---|---|---|---|
| | Competition×Copper×Salinity | 0.00 | 0.00 | 0.0 | 0.9726 | 0.02 |
| | Competition×Food×Salinity | 0.00 | 0.01 | 0.2 | 0.8757 | 0.03 |
| | Copper×Food×Salinity | 0.00 | 0.00 | 0.0 | 0.9978 | 0.01 |
| $r_{pot}$ | (Intercept) | 0.14 | 0.06 | 2.4 | 0.0174 | |
| | **Competition** | **0.11** | **0.05** | **2.1** | **0.0333** | **1.00** |
| | **Copper** | **-0.25** | **0.08** | **3.1** | **0.0016** | **1.00** |
| | Food | -0.00 | 0.05 | 0.0 | 0.9620 | 0.61 |
| | **Salinity** | **0.03** | **0.01** | **3.2** | **0.0016** | **1.00** |
| | Competition×Copper | -0.03 | 0.05 | 0.5 | 0.6141 | 0.40 |
| | Competition×Food | 0.01 | 0.04 | 0.3 | 0.7794 | 0.23 |
| | Competition×Salinity | 0.00 | 0.01 | 0.1 | 0.9215 | 0.25 |
| | Copper×Food | -0.01 | 0.04 | 0.3 | 0.7566 | 0.20 |
| | Copper×Salinity | 0.01 | 0.01 | 1.2 | 0.2175 | 0.75 |
| | Food×Salinity | 0.00 | 0.01 | 0.4 | 0.7059 | 0.25 |
| | Competition×Copper×Food | 0.00 | 0.02 | 0.1 | 0.9307 | 0.01 |
| | Competition×Copper×Salinity | 0.00 | 0.00 | 0.1 | 0.9409 | 0.02 |
| | Competition×Food×Salinity | 0.00 | 0.00 | 0.1 | 0.9218 | 0.01 |
| | Copper×Food×Salinity | 0.00 | 0.00 | 0.0 | 0.9679 | 0.01 |
| Eggs | (Intercept) | 3.98 | 0.28 | 13.9 | <0.0001 | |
| | **Competition** | **-3.39** | **0.38** | **8.7** | **<0.0001** | **1.00** |
| | **Copper** | **1.78** | **0.36** | **4.9** | **<0.0001** | **1.00** |
| | Food | 0.49 | 0.28 | 1.7 | 0.0826 | 0.97 |
| | **Salinity** | **-0.26** | **0.04** | **6.4** | **<0.0001** | **1.00** |
| | **Competition×Copper** | **-2.13** | **0.49** | **4.3** | **<0.0001** | **1.00** |
| | Competition×Food | 0.31 | 0.33 | 0.9 | 0.3561 | 0.66 |
| | **Competition×Salinity** | **0.22** | **0.05** | **4.0** | **0.0001** | **1.00** |
| | Copper×Food | 0.08 | 0.20 | 0.4 | 0.6918 | 0.34 |
| | **Copper×Salinity** | **-0.28** | **0.05** | **5.3** | **<0.0001** | **1.00** |
| | Food×Salinity | 0.01 | 0.03 | 0.3 | 0.7354 | 0.29 |
| | Competition×Copper×Food | 0.01 | 0.12 | 0.1 | 0.9220 | 0.05 |
| | **Competition×Copper×Salinity** | **0.31** | **0.07** | **4.2** | **<0.0001** | **1.00** |
| | Competition×Food×Salinity | -0.01 | 0.03 | 0.2 | 0.8516 | 0.06 |
| | Copper×Food×Salinity | 0.00 | 0.01 | 0.1 | 0.9493 | 0.02 |
| | Competition×Copper×Food×Salinity | 0.00 | 0.00 | 0.0 | 0.9871 | 0.01 |

B. *Brachionus quadridentatus*

| Response | Predictor | Estimate | Standard Error | z | P | RI |
|---|---|---|---|---|---|---|

| | | | | | | |
|---|---|---|---|---|---|---|
| Density | (Intercept) | 209.72 | 14.80 | 14.0 | <0.0001 | |
| | **Competition** | **-179.22** | **19.30** | **9.1** | **<0.0001** | **1.00** |
| | **Copper** | **-159.94** | **17.61** | **8.9** | **<0.0001** | **1.00** |
| | **Food** | **59.09** | **20.96** | **2.8** | **0.0052** | **1.00** |
| | **Salinity** | **-19.20** | **2.23** | **8.5** | **<0.0001** | **1.00** |
| | **Competition×Copper** | **158.42** | **21.21** | **7.3** | **<0.0001** | **1.00** |
| | Competition×Food | 43.92 | 24.78 | 1.8 | 0.0792 | 0.97 |
| | **Competition×Salinity** | **16.95** | **2.89** | **5.8** | **<0.0001** | **1.00** |
| | **Copper×Salinity** | **15.43** | **2.57** | **5.9** | **<0.0001** | **1.00** |
| | Food×Salinity | 5.01 | 3.13 | 1.6 | 0.1129 | 0.90 |
| | **Competition×Copper×Salinity** | **-15.23** | **3.17** | **4.7** | **<0.0001** | **1.00** |
| | Competition×Food×Salinity | -3.08 | 3.68 | 0.8 | 0.4059 | 0.54 |
| | Copper×Food | 10.96 | 19.10 | 0.6 | 0.5690 | 0.46 |
| | Copper×Food×Salinity | -1.06 | 2.52 | 0.4 | 0.6750 | 0.21 |
| | Competition×Copper×Food | -0.72 | 7.02 | 0.1 | 0.9197 | 0.09 |
| | Competition×Copper×Food×Salinity | 0.02 | 0.56 | 0.0 | 0.9666 | 0.01 |
| $r_{obs}$ | (Intercept) | 1.11 | 0.10 | 11.3 | <0.0001 | |
| | **Competition** | **-0.99** | **0.12** | **7.9** | **<0.0001** | **1.00** |
| | Copper | 0.04 | 0.07 | 0.5 | 0.6231 | 0.66 |
| | Food | 0.08 | 0.09 | 0.8 | 0.4012 | 0.89 |
| | **Salinity** | **-0.25** | **0.01** | **19.0** | **<0.0001** | **1.00** |
| | Competition×Copper | 0.01 | 0.05 | 0.1 | 0.8871 | 0.18 |
| | **Competition×Salinity** | **0.23** | **0.02** | **13.6** | **<0.0001** | **1.00** |
| | Competition×Food | -0.05 | 0.10 | 0.5 | 0.5964 | 0.39 |
| | Copper×Food | 0.01 | 0.06 | 0.2 | 0.8123 | 0.18 |
| | Copper×Salinity | 0.00 | 0.01 | 0.0 | 0.9670 | 0.15 |
| | Food×Salinity | 0.00 | 0.01 | 0.1 | 0.9527 | 0.21 |
| | Competition×Copper×Food | 0.00 | 0.04 | 0.1 | 0.9269 | 0.01 |
| | Competition×Copper×Salinity | 0.00 | 0.00 | 0.0 | 0.9785 | 0.01 |
| | Competition×Food×Salinity | 0.00 | 0.01 | 0.1 | 0.9517 | 0.02 |
| | Copper×Food×Salinity | 0.00 | 0.00 | 0.0 | 0.9792 | 0.01 |
| $r_{pot}$ | (Intercept) | 1.11 | 0.10 | 11.0 | <0.0001 | |
| | **Competition** | **-0.93** | **0.13** | **7.2** | **<0.0001** | **1.00** |
| | Copper | 0.07 | 0.09 | 0.8 | 0.4350 | 0.81 |
| | Food | 0.08 | 0.09 | 0.8 | 0.4120 | 0.90 |
| | **Salinity** | **-0.25** | **0.01** | **18.8** | **<0.0001** | **1.00** |
| | Competition×Copper | 0.02 | 0.06 | 0.2 | 0.8060 | 0.23 |
| | Competition×Food | -0.06 | 0.11 | 0.6 | 0.5520 | 0.43 |

| | | | | | | |
|---|---|---|---|---|---|---|
| | **Competition×Salinity** | **0.23** | **0.02** | **13.2** | **<0.0001** | **1.00** |
| | Copper×Food | 0.02 | 0.06 | 0.3 | 0.7960 | 0.21 |
| | Copper×Salinity | 0.00 | 0.01 | 0.2 | 0.8510 | 0.20 |
| | Food×Salinity | 0.00 | 0.01 | 0.1 | 0.9370 | 0.21 |
| | Competition×Copper×Food | 0.00 | 0.04 | 0.1 | 0.9320 | 0.01 |
| | Competition×Food×Salinity | 0.00 | 0.01 | 0.0 | 0.9650 | 0.02 |
| | Competition×Copper×Salinity | 0.00 | 0.00 | 0.0 | 0.9850 | 0.01 |
| | Copper×Food×Salinity | 0.00 | 0.00 | 0.0 | 0.9810 | 0.01 |
| Eggs | (Intercept) | 9.40 | 0.76 | 12.2 | <0.0001 | |
| | **Competition** | **-7.76** | **1.03** | **7.4** | **<0.0001** | **1.00** |
| | **Copper** | **-7.16** | **1.03** | **6.8** | **<0.0001** | **1.00** |
| | Food | 0.69 | 0.57 | 1.2 | 0.2287 | 0.93 |
| | **Salinity** | **-0.85** | **0.11** | **7.6** | **<0.0001** | **1.00** |
| | **Competition×Copper** | **7.02** | **1.44** | **4.8** | **<0.0001** | **1.00** |
| | Competition×Food | 0.09 | 0.47 | 0.2 | 0.8496 | 0.22 |
| | **Competition×Salinity** | **0.79** | **0.15** | **5.0** | **<0.0001** | **1.00** |
| | Copper×Food | -0.16 | 0.46 | 0.3 | 0.7277 | 0.28 |
| | **Copper×Salinity** | **0.75** | **0.15** | **4.8** | **<0.0001** | **1.00** |
| | Food×Salinity | 0.00 | 0.06 | 0.1 | 0.9531 | 0.21 |
| | Competition×Copper×Food | 0.00 | 0.15 | 0.0 | 0.9890 | 0.01 |
| | **Competition×Copper×Salinity** | **-0.71** | **0.22** | **3.2** | **0.0015** | **0.99** |
| | Competition×Food×Salinity | -0.01 | 0.05 | 0.1 | 0.9036 | 0.02 |
| | Copper×Food×Salinity | 0.00 | 0.02 | 0.0 | 0.9928 | 0.01 |

Supplementary Table S3. Output of the linear regression models explaining the effect of copper, food, and salinity, only under competition with the other species, on the response of population density, $r_{obs}$, $r_{pot}$, and total resting eggs in the experiments for (A) *B.* 'Nevada' and (B) *B. quadridentatus*. Estimates, standard errors, z-values, and p-values are reported from multimodel averaging, together with relative importance values (RI). Significant predictors are marked in bold. Interaction terms with AICc<0.001 are not reported.

A. *Brachionus* 'Nevada'

| Response | Predictor | Estimate | Standard Error | z | P | RI |
|---|---|---|---|---|---|---|
| Density | (Intercept) | 24.1517 | 2.2203 | 10.630 | <0.0001 | |
| | Copper | -3.41 | 3.92 | 0.9 | 0.3889 | 0.78 |
| | **Food** | **7.58** | **3.16** | **2.4** | **0.0185** | **1.00** |
| | Salinity | 0.36 | 0.31 | 1.1 | 0.2595 | 0.98 |
| | Copper×Food | 6.24 | 6.24 | 1.0 | 0.3212 | 0.70 |
| | Copper×Salinity | 0.37 | 0.56 | 0.7 | 0.5158 | 0.41 |
| | Food×Salinity | 0.12 | 0.40 | 0.3 | 0.7750 | 0.47 |
| | Copper×Food×Salinity | -0.49 | 0.82 | 0.6 | 0.5516 | 0.30 |
| $r_{obs}$ | (Intercept) | 0.28 | 0.07 | 3.8 | 0.0001 | |
| | **Copper** | **-0.28** | **0.09** | **3.1** | **0.0016** | **1.00** |
| | Food | 0.04 | 0.08 | 0.4 | 0.6745 | 0.84 |
| | Salinity | 0.02 | 0.01 | 1.4 | 0.1634 | 1.00 |
| | Copper×Food | 0.00 | 0.03 | 0.1 | 0.8868 | 0.15 |
| | Copper×Salinity | 0.01 | 0.01 | 0.9 | 0.3455 | 0.62 |
| | Food×Salinity | 0.01 | 0.01 | 1.0 | 0.3182 | 0.62 |
| $r_{pot}$ | (Intercept) | 0.25 | 0.06 | 3.7 | 0.0002 | |
| | **Copper** | **-0.25** | **0.06** | **3.7** | **0.0002** | **1.00** |
| | Food | 0.04 | 0.08 | 0.4 | 0.6541 | 0.80 |
| | **Salinity** | **0.03** | **0.01** | **2.6** | **0.0090** | **1.00** |
| | Copper×Food | 0.00 | 0.03 | 0.1 | 0.9491 | 0.14 |
| | Copper×Salinity | 0.00 | 0.01 | 0.5 | 0.6409 | 0.32 |
| | Food×Salinity | 0.01 | 0.01 | 0.9 | 0.3454 | 0.58 |
| Eggs | (Intercept) | 0.29 | 0.15 | 1.9 | 0.0612 | |
| | Copper | -0.06 | 0.14 | 0.4 | 0.6634 | 0.37 |
| | Food | 0.02 | 0.10 | 0.2 | 0.8472 | 0.29 |
| | Salinity | -0.01 | 0.02 | 0.5 | 0.6387 | 0.38 |

| Response | Predictor | Estimate | Standard Error | z | P | RI |
|---|---|---|---|---|---|---|
| | Copper×Food | 0.02 | 0.09 | 0.2 | 0.8693 | 0.04 |
| | Copper×Salinity | 0.00 | 0.01 | 0.1 | 0.9160 | 0.04 |
| | Food×Salinity | 0.00 | 0.01 | 0.0 | 0.9622 | 0.02 |

B. *Brachionus quadridentatus*

| Response | Predictor | Estimate | Standard Error | z | P | RI |
|---|---|---|---|---|---|---|
| Density | (Intercept) | 30.37 | 1.46 | 19.9 | <0.0001 | |
| | **Copper** | **-7.41** | **2.07** | **3.4** | **0.0006** | **1.00** |
| | **Food** | **14.90** | **2.07** | **6.9** | **<0.0001** | **1.00** |
| | **Salinity** | **-2.18** | **0.22** | **9.4** | **<0.0001** | **1.00** |
| | **Copper×Food** | **22.03** | **2.92** | **7.2** | **<0.0001** | **1.00** |
| | **Copper×Salinity** | **1.04** | **0.31** | **3.2** | **0.0015** | **1.00** |
| | **Food×Salinity** | **1.77** | **0.31** | **5.4** | **<0.0001** | **1.00** |
| | **Copper×Food×Salinity** | **-2.71** | **0.44** | **5.9** | **<0.0001** | **1.00** |
| $r_{obs}$ | (Intercept) | 0.24 | 0.03 | 7.7 | <0.0001 | |
| | **Copper** | **-0.18** | **0.04** | **4.2** | **<0.0001** | **1.00** |
| | **Food** | **0.40** | **0.04** | **9.2** | **<0.0001** | **1.00** |
| | **Salinity** | **-0.03** | **0.00** | **5.8** | **<0.0001** | **1.00** |
| | **Copper×Food** | **0.53** | **0.06** | **8.5** | **<0.0001** | **1.00** |
| | **Copper×Salinity** | **0.03** | **0.01** | **4.3** | **<0.0001** | **1.00** |
| | **Food×Salinity** | **0.02** | **0.01** | **2.9** | **0.0042** | **1.00** |
| | **Copper×Food×Salinity** | **-0.05** | **0.01** | **5.4** | **<0.0001** | **1.00** |
| $r_{pot}$ | (Intercept) | 0.25 | 0.04 | 6.0 | <0.0001 | |
| | Copper | 0.01 | 0.06 | 0.1 | 0.9010 | 0.90 |
| | **Food** | **0.31** | **0.06** | **4.9** | **<0.0001** | **1.00** |
| | **Salinity** | **-0.03** | **0.01** | **4.4** | **<0.0001** | **1.00** |
| | **Copper×Food** | **0.28** | **0.12** | **2.3** | **0.0198** | **1.00** |
| | Copper×Salinity | 0.00 | 0.01 | 0.4 | 0.6599 | 0.48 |
| | Food×Salinity | 0.01 | 0.01 | 0.5 | 0.5894 | 0.46 |
| | Copper×Food×Salinity | -0.01 | 0.02 | 0.7 | 0.4945 | 0.36 |
| Eggs | (Intercept) | 1.24 | 0.30 | 4.0 | 0.0001 | |
| | Copper | 0.02 | 0.18 | 0.1 | 0.9050 | 0.32 |
| | Food | 0.11 | 0.52 | 0.2 | 0.8300 | 1.00 |
| | Salinity | 0.01 | 0.04 | 0.2 | 0.8590 | 0.80 |
| | Copper×Food | -0.06 | 0.22 | 0.3 | 0.7800 | 0.12 |
| | Copper×Salinity | 0.00 | 0.02 | 0.1 | 0.9160 | 0.06 |
| | Food×Salinity | -0.09 | 0.08 | 1.1 | 0.2900 | 0.64 |

| | | | | | |
|---|---|---|---|---|---|
| Copper×Food×Salinity | 0.00 | 0.01 | 0.0 | 0.9690 | 0.01 |

Supplementary Table S4. Output of the linear regression models testing the differences between species on population density, $r_{obs}$, $r_{pot}$, and total diapausing eggs in the experiments on single-Species cultures with varying levels of the effect of salinity, food, and copper. Estimates, standard errors, z-values, and p-values are reported from multimodel averaging, together with relative importance values (RI). Significant predictors are marked in bold. Interaction terms with AICc<0.001 are not reported.

| Response | Predictor | Estimate | Standard Error | z | P | RI |
|---|---|---|---|---|---|---|
| Density | (Intercept) | 12.10 | 14.07 | 0.8 | 0.40 | |
| | Copper | -6.59 | 17.07 | 0.4 | 0.71 | 1.00 |
| | Food | 14.24 | 17.81 | 0.8 | 0.43 | 1.00 |
| | Salinity | 2.17 | 2.12 | 1.0 | 0.31 | 1.00 |
| | **Species** | **195.55** | **19.91** | **9.7** | **<0.00** | **1.00** |
| | Copper×Food | 2.92 | 14.67 | 0.2 | 0.84 | 0.37 |
| | Copper×Salinity | -0.65 | 2.58 | 0.2 | 0.80 | 1.00 |
| | **Copper×Species** | **-151.08** | **22.64** | **6.5** | **<0.00** | **1.00** |
| | Food×Salinity | 2.13 | 2.65 | 0.8 | 0.43 | 0.87 |
| | Food×Species | -40.72 | 25.21 | 1.6 | 0.11 | 0.95 |
| | **Salinity×Species** | **-21.17** | **2.92** | **7.1** | **<0.00** | **1.00** |
| | Copper×Food×Salinity | -0.78 | 2.24 | 0.3 | 0.73 | 0.16 |
| | Copper×Food×Species | 3.50 | 12.23 | 0.3 | 0.78 | 0.13 |
| | **Copper×Salinity×Species** | **15.96** | **3.31** | **4.7** | **0.00** | **1.00** |
| | Food×Salinity×Species | 2.48 | 3.51 | 0.7 | 0.48 | 0.45 |
| | Copper×Food×Salinity×Species | -0.03 | 0.63 | 0.0 | 0.96 | 0.01 |
| $r_{obs}$ | (Intercept) | 0.04 | 0.10 | 0.4 | 0.69 | |
| | Copper | -0.12 | 0.11 | 1.1 | 0.28 | 0.64 |
| | Food | 0.02 | 0.08 | 0.2 | 0.84 | 0.39 |
| | **Salinity** | **0.03** | **0.01** | **2.2** | **0.02** | **1.00** |
| | **Species** | **1.05** | **0.13** | **7.9** | **<0.00** | **1.00** |
| | Copper×Food | -0.07 | 0.11 | 0.6 | 0.56 | 0.07 |
| | Copper×Salinity | 0.01 | 0.02 | 0.6 | 0.54 | 0.19 |
| | Copper×Species | 0.20 | 0.13 | 1.5 | 0.14 | 0.39 |
| | Food×Salinity | 0.00 | 0.02 | 0.1 | 0.94 | 0.09 |
| | Food×Species | -0.03 | 0.12 | 0.3 | 0.78 | 0.09 |
| | **Salinity×Species** | **-0.28** | **0.02** | **15.6** | **<0.00** | **1.00** |
| | Copper×Food×Salinity | -0.01 | 0.04 | 0.2 | 0.86 | 0.01 |
| | Copper×Food×Species | 0.01 | 0.22 | 0.0 | 0.96 | 0.01 |
| | Copper×Salinity×Species | -0.03 | 0.03 | 0.9 | 0.35 | 0.04 |
| | Food×Salinity×Species | 0.01 | 0.04 | 0.4 | 0.68 | 0.01 |
| $r_{pot}$ | (Intercept) | 0.07 | 0.10 | 0.6 | 0.53 | |
| | Copper | -0.09 | 0.11 | 0.8 | 0.42 | 0.69 |
| | Food | 0.01 | 0.05 | 0.1 | 0.92 | 0.39 |
| | **Salinity** | **0.03** | **0.01** | **2.4** | **0.02** | **1.00** |
| | **Species** | **1.04** | **0.14** | **7.4** | **0.00** | **1.00** |
| | Copper×Food | -0.01 | 0.04 | 0.2 | 0.87 | 0.08 |
| | Copper×Salinity | 0.00 | 0.01 | 0.2 | 0.84 | 0.19 |
| | Copper×Species | 0.11 | 0.15 | 0.7 | 0.47 | 0.48 |
| | Food×Salinity | 0.00 | 0.01 | 0.0 | 0.97 | 0.09 |
| | Food×Species | 0.00 | 0.04 | 0.1 | 0.94 | 0.09 |
| | **Salinity×Species** | **-0.29** | **0.02** | **15.4** | **<0.00** | **1.00** |
| | Copper×Food×Salinity | 0.00 | 0.00 | 0.0 | 0.99 | 0.01 |
| | Copper×Food×Species | 0.00 | 0.01 | 0.0 | 0.99 | 0.01 |
| | Copper×Salinity×Species | 0.00 | 0.01 | 0.2 | 0.8777 | 0.05 |
| | Food×Salinity×Species | 0.00 | 0.00 | 0.0 | 0.9867 | 0.01 |
| Eggs | (Intercept) | 4.08 | 0.76 | 5.3 | <0.00 | |

| | | | | | |
|---|---|---|---|---|---|
| Copper | 1.84 | 0.99 | 1.8 | 0.07 | 1.00 |
| Food | 0.70 | 0.63 | 1.1 | 0.27 | 0.89 |
| **Salinity** | **-0.27** | **0.11** | **2.4** | **0.02** | **1.00** |
| **Species** | **5.39** | **1.00** | **5.3** | **0.00** | **1.00** |
| Copper×Food | -0.05 | 0.35 | 0.1 | 0.89 | 0.21 |
| Copper×Salinity | -0.28 | 0.15 | 1.9 | 0.06 | 1.00 |
| **Copper×Species** | **-9.07** | **1.39** | **6.4** | **<0.00** | **1.00** |
| Food×Salinity | 0.03 | 0.07 | 0.4 | 0.71 | 0.28 |
| Food×Species | -0.10 | 0.40 | 0.2 | 0.81 | 0.23 |
| **Salinity×Species** | **-0.60** | **0.15** | **4.0** | **0.00** | **1.00** |
| Copper×Food×Salinity | 0.00 | 0.02 | 0.0 | 0.99 | 0.01 |
| Copper×Food×Species | -0.01 | 0.17 | 0.1 | 0.96 | 0.01 |
| **Copper×Salinity×Species** | **1.03** | **0.21** | **4.8** | **0.00** | **1.00** |
| Food×Salinity×Species | 0.00 | 0.03 | 0.0 | 0.97 | 0.01 |

Supplementary Table S5. Output of the linear regression models testing the differences between species on population density, $r_{obs}$, $r_{pot}$, and total diapausing eggs in the experiments in competition with varying levels of the effect of copper, food, and salinity. Estimates, standard errors, z-values, and p-values are reported from multimodel averaging, together with relative importance values (RI). Significant predictors are marked in bold. Interaction terms with AICc<0.001 are not reported.

| Response | Predictor | Estimate | Standard Error | z | P | RI |
|---|---|---|---|---|---|---|
| Density | (Intercept) | 26.64 | 1.68 | 15.6 | <0.00 | |
| | **Copper** | **-8.62** | **2.14** | **4.0** | **0.00** | **1.00** |
| | **Food** | **-13.40** | **2.32** | **5.7** | **<0.00** | **1.00** |
| | Salinity | -0.01 | 0.25 | 0.0 | 0.96 | 1.00 |
| | Species | 3.24 | 2.25 | 1.4 | 0.16 | 1.00 |
| | **Copper×Food** | **18.10** | **2.61** | **6.8** | **<0.00** | **1.00** |
| | **Copper×Salinity** | **1.15** | **0.31** | **3.6** | **0.00** | **1.00** |
| | Copper×Species | 1.45 | 2.42 | 0.6 | 0.55 | 0.52 |
| | **Food×Salinity** | **0.94** | **0.35** | **2.6** | **0.01** | **1.00** |
| | Food×Species | 1.07 | 3.00 | 0.4 | 0.72 | 1.00 |
| | **Salinity×Species** | **-2.08** | **0.34** | **6.1** | **<0.00** | **1.00** |
| | **Copper×Food×Salinity** | **-2.17** | **0.39** | **5.5** | **<0.00** | **1.00** |
| | Copper×Food×Species | 0.29 | 1.51 | 0.2 | 0.85 | 0.12 |
| | Copper×Salinity×Species | -0.15 | 0.33 | 0.5 | 0.65 | 0.27 |
| | Food×Salinity×Species | 0.45 | 0.46 | 1.0 | 0.34 | 0.63 |
| | Copper×Food×Salinity×Species | -0.02 | 0.18 | 0.1 | 0.91 | 0.02 |
| $r_{obs}$ | (Intercept) | 0.31 | 0.06 | 5.0 | <0.00 | |
| | **Copper** | **-0.31** | **0.08** | **3.7** | **0.00** | **1.00** |
| | Food | -0.06 | 0.09 | 0.7 | 0.45 | 1.00 |
| | Salinity | 0.01 | 0.01 | 1.3 | 0.19 | 1.00 |
| | Species | -0.09 | 0.08 | 1.1 | 0.28 | 1.00 |
| | Copper×Food | 0.00 | 0.12 | 0.0 | 0.99 | 1.00 |
| | Copper×Salinity | 0.01 | 0.01 | 1.2 | 0.24 | 0.91 |
| | Copper×Species | 0.17 | 0.12 | 1.4 | 0.16 | 1.00 |
| | Food×Salinity | 0.02 | 0.01 | 1.4 | 0.17 | 0.93 |
| | **Food×Species** | **-0.29** | **0.14** | **2.1** | **0.04** | **1.00** |
| | **Salinity×Species** | **-0.04** | **0.01** | **2.9** | **0.00** | **1.00** |
| | Copper×Food×Salinity | 0.01 | 0.02 | 0.3 | 0.75 | 0.67 |
| | Copper×Food×Species | 0.40 | 0.24 | 1.7 | 0.10 | 0.95 |
| | Copper×Salinity×Species | 0.01 | 0.02 | 0.3 | 0.75 | 0.68 |
| | Food×Salinity×Species | -0.01 | 0.02 | 0.4 | 0.69 | 0.86 |
| | Copper×Food×Salinity×Species | -0.04 | 0.04 | 1.0 | 0.34 | 0.54 |
| $r_{pot}$ | (Intercept) | 0.25 | 0.06 | 4.2 | <0.00 | |
| | **Copper** | **-0.25** | **0.06** | **4.0** | **0.00** | **1.00** |
| | Food | -0.05 | 0.09 | 0.6 | 0.58 | 1.00 |
| | **Salinity** | **0.03** | **0.01** | **3.1** | **0.00** | **1.00** |
| | Species | -0.01 | 0.08 | 0.2 | 0.85 | 1.00 |
| | Copper×Food | 0.03 | 0.08 | 0.4 | 0.68 | 1.00 |
| | Copper×Salinity | 0.00 | 0.01 | 0.4 | 0.68 | 0.37 |
| | **Copper×Species** | **0.29** | **0.08** | **3.4** | **0.00** | **1.00** |
| | Food×Salinity | 0.02 | 0.01 | 1.2 | 0.24 | 0.76 |
| | Food×Species | -0.23 | 0.12 | 1.9 | 0.05 | 1.00 |
| | **Salinity×Species** | **-0.05** | **0.01** | **4.6** | **0.00** | **1.00** |
| | Copper×Food×Salinity | 0.00 | 0.01 | 0.0 | 0.97 | 0.08 |
| | Copper×Food×Species | 0.16 | 0.12 | 1.3 | 0.20 | 0.79 |
| | Copper×Salinity×Species | 0.00 | 0.01 | 0.3 | 0.80 | 0.15 |
| | Food×Salinity×Species | -0.01 | 0.02 | 0.8 | 0.42 | 0.53 |

| | | | | | | |
|---|---|---|---|---|---|---|
| | Copper×Food×Salinity×Species | 0.00 | 0.01 | 0.1 | 0.89 | 0.02 |
| Eggs | (Intercept) | 0.30 | 0.24 | 1.2 | 0.21 | |
| | Copper | -0.14 | 0.22 | 0.6 | 0.54 | 0.45 |
| | Food | 0.18 | 0.35 | 0.5 | 0.62 | 1.00 |
| | Salinity | -0.01 | 0.03 | 0.2 | 0.83 | 0.90 |
| | **Species** | **1.02** | **0.26** | **3.8** | **0.00** | **1.00** |
| | Copper×Food | 0.03 | 0.34 | 0.1 | 0.93 | 0.12 |
| | Copper×Salinity | 0.03 | 0.04 | 0.8 | 0.40 | 0.12 |
| | Copper×Species | 0.23 | 0.32 | 0.7 | 0.48 | 0.13 |
| | Food×Salinity | -0.06 | 0.05 | 1.3 | 0.19 | 0.68 |
| | Food×Species | -0.49 | 0.40 | 1.2 | 0.22 | 1.00 |
| | Salinity×Species | 0.01 | 0.05 | 0.1 | 0.92 | 0.32 |
| | Copper×Food×Salinity | -0.01 | 0.08 | 0.2 | 0.86 | 0.01 |
| | Copper×Food×Species | -0.85 | 0.47 | 1.8 | 0.08 | 0.03 |
| | Food×Salinity×Species | -0.12 | 0.07 | 1.6 | 0.11 | 0.14 |
| | Copper×Salinity×Species | 0.01 | 0.08 | 0.1 | 0.94 | 0.01 |
